# A comprehensive scoping review on transvenous temporary pacing therapy

**DOI:** 10.1007/s12471-019-01307-x

**Published:** 2019-08-07

**Authors:** F. V. Y. Tjong, U. W. de Ruijter, N. E. G. Beurskens, R. E. Knops

**Affiliations:** Heart Centre, Department of Experimental and Clinical Cardiology, Amsterdam University Medical Centre, Location AMC, Amsterdam, The Netherlands

**Keywords:** Transvenous temporary pacing, Arrhythmia, Indications, Access site, Complications

## Abstract

Transvenous temporary cardiac pacing therapy (TV-TP) is widely used to treat life-threatening arrhythmias. Yet aggregated evidence on TV-TP is limited. We conducted a systematic scoping review to evaluate indications, access routes and complications of TV-TP, as well as permanent pacemaker therapy (PPM) following TV-TP. Clinical studies concerning TV-TP were identified in Ovid MEDLINE. Case studies and studies lacking complication rates were excluded. To assess complication incidence over time, differences in mean complication rates between 10-year intervals since the introduction of TV-TP were evaluated. We identified 1398 studies, of which 32 were included, effectively including 4546 patients. Indications varied considerably; however TV-TP was most commonly performed in atrioventricular block (62.7%). The preferred site of access was the femoral vein (47.2%). The mean complication rate was 36.7%, of which 10.2% were considered serious. The incidence of complications decreased significantly between 10-year interval groups, but remained high in the most recent time period (22.9%) (analysis of variance; *p* < 0.001). PPM was required in 64.2% of cases following TV-TP. Atrioventricular block was the primary indication for TV-TP; however indications varied widely. The femoral vein was the most frequent approach. Complications are common in patients undergoing TV-TP. Although a decrease has been observed since its introduction, the clinical burden remains significant. The majority of patients who underwent TV-TP required PPM therapy.

**Electronic supplementary material:**

The online version of this article (10.1007/s12471-019-01307-x) contains supplementary material, which is available to authorized users.

## Introduction

Transvenous temporary cardiac pacing (TV-TP) is a potentially life-saving therapy in patients with haemodynamically compromising arrhythmias [1]. TV-TP therapy is primarily indicated for the treatment of symptomatic bradycardia and various types of (reversible) symptomatic heart block [2]. In addition, TV-TP therapy may be used as a bridge to permanent cardiac pacing when permanent pacing is not immediately indicated or available, or when a permanent pacemaker cannot be implanted. Other possible indications include myocardial infarction, and injury to the conduction system following (non-)cardiac surgery (e.g. transcatheter aortic valve implantation, coronary artery bypass grafting, valve surgery) [2–4].

Since the first report on its successful use by Tancredi et al. in 1967, the main concept of TV-TP therapy has not changed considerably, even though patients are at risk for procedure-related complications resulting in patient morbidity and mortality [5]. Observed complications are related to the transvenous lead (e.g. lead dislodgement, lead malfunction, cardiac perforation) or related to the venous access and the necessity for immobilisation (e.g. bleeding, infection, thrombosis and delirium, especially in the elderly population) [6–9]. These complications result in re-intervention and prolonged hospitalisation. Moreover, it is not uncommon for the pacing indication to persist after implantation of a temporary transvenous pacemaker, requiring a second procedure to implant a permanent pacemaker (PPM), often performed more than a week after implantation of the temporary pacemaker [10]. Previous studies have investigated complication rates, yet the reported results are inconsistent and conflicting. Reported complication rates of TV-TP therapy are high, ranging from 10 to 60%, with an average of 26.5% [8–11]. Consequently, current ESC guidelines recommend that TV-TP therapy should be avoided or applied as briefly as possible [12].

Available evidence on indications, approach and complications of TV-TP therapy is limited. In addition, the need for PPM therapy following TV-TP has not yet been elucidated, but is of high clinical relevance. A substantial number of studies date back to before 1980, and it is unclear how these results translate to current clinical practice. To understand the current risks and benefits of TV-TP and the potential need for alternatives, a comprehensive review of the available evidence on TV-TP is essential. Therefore, a scoping review was performed in order to give an up-to-date overview on indications, access route and complications of TV-TP as well as the need for subsequent permanent pacemaker implantation.

## Methods

A scoping review of studies reporting TV-TP indications, approaches, complications and need for PPM was conducted. Due to the scoping nature, our protocol was not eligible for assessment by PRISMA guidelines, nor for inclusion in PROSPERO. A prospectively designed protocol which defined in- and exclusion criteria, search strategy and definitions of complications and indications was developed.

### Search

A systematic literature search for relevant articles published until February 2019 was carried out in the Ovid MEDLINE database. The search strategy, including terms and limits, was determined in collaboration with a medical information specialist (see Electronic Supplementary Material, Supplement 1).

Studies were eligible if they included adults requiring TV-TP therapy for bradycardia, inadequate escape rhythm or asystole. Case series with fewer than ten patients were excluded from the analysis, as were studies concerning transoesophageal, transthoracic, transcutaneous or atrial pacing. Studies without mention of complication rates were excluded. Studies were restricted to those published in English or Dutch and those conducted in humans. Studies with specific inclusion criteria regarding either patient category or complications were reviewed separately from consecutive patient series in order not to confound mean complication rates.

### *Data extraction*

Two investigators (F.V.Y. Tjong, U.W. de Ruijter) independently appraised all studies and consequently extracted all relevant data from the selected studies. Differing appraisals were resolved by means of consensus. The following data were extracted: patient characteristics, indication for and duration of TV-TP therapy, definition of complications, complication rates, mortality, re-interventions, site of access and subsequent PPM implantation. A pre-specified data extraction sheet was included in the protocol.

### Indications

Ten groups of indications for TV-TP therapy were defined: cardiac arrest, atrioventricular block, sinus node disease, acute myocardial infarction, permanent pacemaker failure, bradycardia, prophylactic or periprocedural use, overdrive suppression, drug toxicity and other or unknown (Tab. 1). Groups were mutually exclusive.

**Table 1:** Indications for transvenous temporary pacing therapy (4546 patients)

	*n* = 4546
1. Asystole or cardiac arrest	1.1%
2. Atrioventricular block (AVB all degrees, AF with slow rate)	62.7%
3. Sinus node disease	6.7%
4. Acute myocardial infarction (underlying rhythm not specified)	11.4%
5. Permanent pacemaker failure	4.4%
6. Bradycardia (sinus bradycardia, sinus pause, sinus arrest, AV nodal escape rhythm)	4.9%
7. Prophylactic or periprocedural (prophylactic, diagnostic, required for procedure)	2.7%
8. Overdrive suppression (VPC overdrive, VT overdrive)	2.3%
9. Drug toxicity (medication washout, drug overdose, digitalis intoxication)	2.4%
10. Other or unknown	1.5%

*AVB* atrioventricular block, *AF* atrial fibrillation, *AV* atrioventricular,* VPC* ventricular premature contraction, *VT* ventricular tachycardia

A major indication for TV-TP therapy is in the setting of acute myocardial infarction, especially in previous decades [13–15]. We strived to categorise this indication according to underlying arrhythmia if data were provided. In case this was not reported, the indication was categorised by default as acute myocardial infarction.

### Complications

Complications were categorised in eight main groups: complicated access, cardiac perforation, device complications, infection, arrhythmia, thrombotic event, procedure-related death and other or unknown (Tab. 2). Pericardial effusion without signs of systemic infection was considered a cardiac perforation. Pericardial effusion with signs of infection was considered to be pericarditis. Malpacing and malsensing were considered to be TV-TP failure.

**Table 2:** Complications reported

	Before 1980	1980–1989	1990–1999	2000–2009	2010–2019	Total
Number of patients (studies)	504 (4)	1981 (9)	291 (4)	890 (6)	880 (9)	4546 (32)
*1. Complicated access*	0.4%	1.0%	2.1%	2.8%	4.2%	2.0%
Minor bleed access site	0.2%	0.0%	0.0%	1.7%	1.0%	0.5%
Arterial puncture	0.0%	0.7%	1.0%	0.5%	0.3%	0.5%
Brachial plexus injury	0.0%	0.0%	0.3%	0.0%	0.0%	0.0%
Pneumothorax	0.0%	0.1%	0.7%	0.2%	0.1%	0.1%
Excessive bleed access site	0.2%	0.2%	0.0%	0.4%	2.7%	0.7%
*2. Cardiac perforation*	2.0%	1.8%	0.5%	2.1%	0.7%	1.6%
*3. Device complications*	21.0%	27.1%	25.1%	14.7%	12.9%	21.1%
TP lead dislodgement	11.5%	2.6%	14.1%	3.1%	3.4%	4.6%
TP failure	8.6%	13.3%	0.7%	6.2%	7.7%	9.5%
Multiple attempts	0.3%	0.0%	10.3%	5.4%	0.9%	1.9%
Re-intervention	0.6%	11.2%	0.0%	0.0%	0.9%	5.1%
*4. Infections*	2.8%	5.7%	5.2%	5.0%	3.6%	4.8%
Fever (>38 °C)	0.4%	0.3%	0.0%	1.2%	0.0%	0.4%
Phlebitis	1.4%	1.4%	0.0%	0.0%	0.2%	0.8%
Local wound infection	0.8%	1.0%	1.0%	1.5%	2.4%	1.3%
Sepsis or systemic infection	0.2%	0.2%	4.2%	2.1%	0.9%	1.0%
Pericarditis	0.0%	2.8%	0.0%	0.2%	0.1%	1.3%
*5. Arrhythmia*	4.0%	10.2%	5.5%	1.9%	0.5%	5.7%
VT during insertion	3.0%	3.1%	0.0%	0.0%	0.0%	1.7%
Arrhythmias	1.0%	7.2%	5.5%	1.9%	0.5%	4.0%
*6. Thrombotic event*	0.4%	0.2%	0.0%	0.7%	0.5%	0.3%
Deep venous thrombosis	0.0%	0.0%	0.0%	0.7%	0.3%	0.2%
Pulmonary embolism	0.4%	0.2%	0.0%	0.0%	0.1%	0.1%
*7. Death due to TP*	0.4%	0.0%	0.2%	0.7%	0.2%	0.2%
*8. Other complications*	0.2%	1.6%	0.9%	1.0%	0.3%	1.0%
Total	31.2%	47.6%	39.5%	28.1%	22.9%	36.7%

*TP* temporary pacing*, VT* ventricular tachycardia

According to current guidelines, complications are considered serious when (re-)intervention is required [12]. (Re-)interventions following TV-TP were not systematically documented in all published studies. We considered the following complications to warrant (re-)intervention: sepsis, cardiac perforation, excessive bleeding at access site and pulmonary embolism. We therefore defined these complications—in addition to procedure-related death—as serious complications. Procedure-related ventricular fibrillation and asystole were not included as serious complications as these usually arise during device insertion and can be corrected at this stage.

In order to assess a trend in complication rates since the introduction of TV-TP therapy we reviewed mean complication rates over the years in 10-year intervals, based on year of publication.

### Statistical analysis

Continuous data were described as weighted means ± standard deviations (SD) and categorical data as weighted percentages. Statistical analysis was performed using SPSS (Version 23, 2015) and Microsoft Excel 2010. One-way analysis of variance (ANOVA) was used to assess significant changes in complication rates between the 10-year intervals.

## Results

**Fig. 1:**
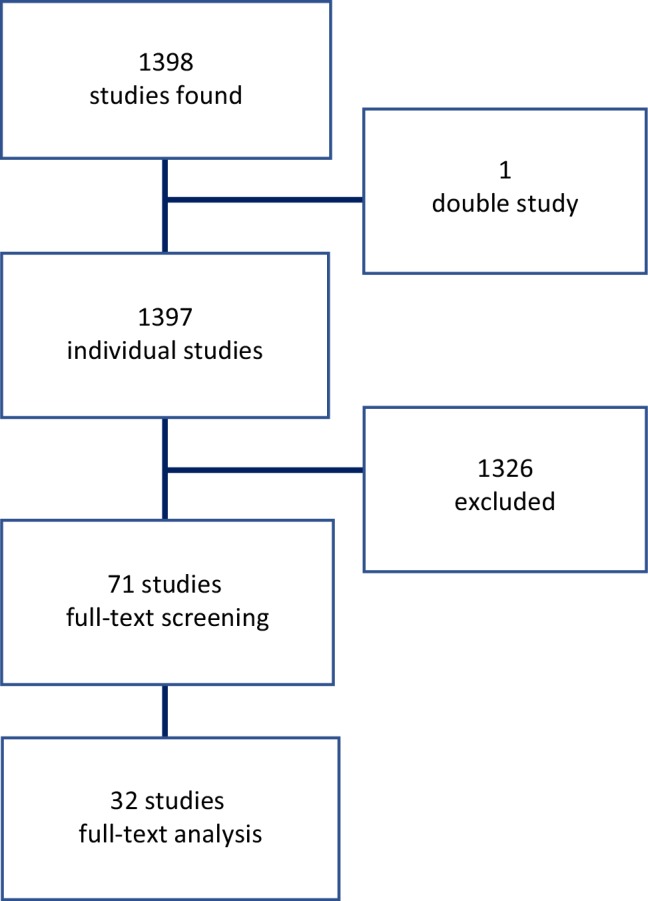
Flowchart showing the selection process

The search strategy identified a total of 1398 individual studies. After the first screening, 71 potentially eligible studies were reviewed in full-text form. An additional 33 studies proved insufficient to answer our main objectives and were excluded. The selection process is displayed in Fig. 1. A total of 32 original papers with series of consecutive patients requiring TV-TP met the inclusion criteria with a total of 4546 individual patients (mean age 71.3 years, 61.5% male) (Fig. 1). One additional study is highlighted separately, because it was not eligible for inclusion in our data analysis, yet it possessed valuable information on the use and outcomes of TV-TP therapy in a very large US patient cohort (*n* = 360,233) [16].

### Indications

The most important indication for TV-TP was atrioventricular block (62.7%), followed by acute myocardial infarction with no specified underlying rhythm (11.4%) and sinus node disease (6.7%) (Tab. 1). Other or unknown indications (1.5%) comprise atrial flutter, torsades des pointes, electrolyte imbalance and unknown indications. In 40.8% of cases TV-TP therapy was required in the setting of acute myocardial infarction, resulting in AV block or no specified underlying rhythm, as documented in 26 out of 32 studies (*n* = 4150).

### *Access site*

In 25 out of 31 studies (*n* = 3109) the access site of TV-TP was described. The most frequently used approach was through the femoral vein (47.2%), followed by the subclavian vein (25.4%). Other reported access sites were the internal jugular vein (12.0%), antecubital or antebrachial vein (10.4%), cephalic vein (3.5%) and the brachial vein (0.5%). In 0.1% of cases the access site was reported as other or unknown.

### Complications

**Fig. 2:**
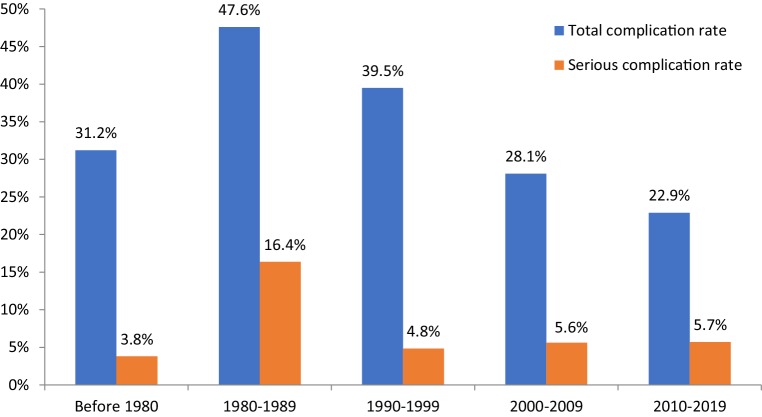
Bar chart demonstrating the complication rates (%) since the introduction of temporary transvenous pacing therapy in 1967. Ten-year intervals, based on year of publication, are shown on the *x-axis*. The total complication rate and serious complication rate are shown in percentages on the *y-axis*

The total complication rate ranged from 0.8 to 94.2% (Tab. 3). The weighted mean complication rate of all included studies was 36.7%, of which 10.2% were considered serious. The mean complication rate improved from 31.2% in the period before 1980 to 22.9% between 2010 and 2019 (Fig. 2). The mean total complication rates between the 10-year intervals showed a significant decrease (ANOVA; *p*-value <0.001). The most common complication by far concerned the device (21.0%), followed by arrhythmias (5.6%) and infection (4.8%). Among the device complications, TV-TP failure (including malsensing/malpacing) was the most frequent (9.5%), followed by the need for a re-intervention to place the TV-TP lead (5.4%), TV-TP lead dislodgement without requiring a re-intervention (4.5%), and multiple placement attempts (1.9%). The most common serious complication was re-intervention (5.4%) followed by cardiac perforation (1.6%). Other or unknown complications comprised atrial flutter, diaphragmatic stimulation, high pacing threshold and suspected pneumonia. Mortality was reported in 17 out of 31 studies (*n* = 3144) with a weighted mean of 14.5%, as can be seen in Tab. 3. In 15 studies the duration of TV-TP therapy was reported (*n* = 2665), resulting in the need for prolonged hospitalisation with a mean of 11.2 days.

**Table 3:** Complication and mortality rates per study

Year of publication	First author	Number of cases	Complication rate	Serious complication rate	Mortality
1967	Tancredi [5]	91	35.3%	6.6%	2.2%
1971	Javier [17]	200	5.0%	0.0%	0.0%
1973	Lumia[18]	113	94.2%	9.3%	1.8%
1973	Weinstein [19]	100	8.0%	2.0%	29.0%
1981	Lang [20]	44	52.4%	0.0%	N/A
1982	Austin [8]	100	85.0%	12.4%	4.0%
1983	Hynes [21]	1022	46.9%	22.7%	17.6%
1983	Papasteriadis [22]	42	7.2%	0.0%	N/A
1983	Paterson [23]	117	22.0%	0.8%	N/A
1985	Chin [24]	111	81.9%	3.6%	15.3%
1987	Abinader [25]	339	37.9%	0.9%	N/A
1987	Seng [26]	44	2.3%	2.3%	50%
1989	Jowett [27]	162	19.6%	1.8%	32.3%
1993	Liu [28]	53	56.6%	1.9%	N/A
1993	Rashid [29]	50	10.0%	4.0%	30.0%
1996	Murphy [30]	168	44.5%	6.0%	34.0%
1997	Ferguson [31]	20	15.0%	5.0%	N/A
2003	Betts [32]	111	64.8%	11.7%	N/A
2003	De Cock A [33]	42	26.3%	0.0%	N/A
2003	De Cock B [34]	36	44.4%	0.0%	N/A
2004	Ayerbe [35]	530	22.6%	4.8%	6.4%
2004	Birkhahn [36]	117	21.2%	7.4%	23.1%
2007	Sodeck [37]	54	5.6%	3.7%	N/A
2010	Garcia [38]	47	23.5%	6.4%	N/A
2011	Bono [39]	182	39.0%	13.8%	2.7%
2012	Deftereos [40]	25	12.0%	0.0%	0.0%
2012	Björnstad [41]	50	51.8%	5.7%	16.0%
2013	Pinneri [42]	106	24.4%	6.5%	N/A
2014	Palmisano [43]	79	11.4%	0.0%	2.5%
2015	Shah [44]	122	0.8%	0.0%	N/A
2016	Ferri [45]	203	21.3%	2.0%	N/A
2018	El Nasasra [46]	66	9.0%	4.5%	N/A

### Permanent pacing therapy

In 18 out of 32 studies (*n* = 3017) the need for PPM placement following TV-TP was documented. In 64.2% of patients, permanent pacing was required after TV-TP therapy. No data were provided on the time until PPM placement.

### Large observational study on TV-TP therapy in the United States

In 2019, the largest study to date on the use of TV-TP therapy and outcomes related to it was published on a US patient cohort from the National Inpatient Sample (NIS) database [16]. It reviewed over 360,000 patient records between 2004 and 2014. Due to the methodological limitations of this NIS patient cohort and the lack of definite TV-TP-related complications, this study could not be included in our analysis. However, the findings of this study are interesting and worth reporting. In total 360,223 patients underwent TV-TP therapy. The annual rate of temporary transvenous pacemaker insertion remained stable between 10 and 12 per 100,000 US population throughout the decade. The in-hospital mortality was 14.1%. Potential procedural complications linked to TV-TP were pericardial tamponade in 0.6%, pneumothorax in 0.9% and non-pericardial bleeding in 2.4% of patients, leading to a total complication rate of 3.9%. In a multivariate logistic regression model several factors were associated with an increased risk for cardiac tamponade: female sex [odds ratio (OR) 1.33; 95% confidence interval (CI) 1.09–1.64; *p* = 0.005], in-hospital cardiac arrest (OR 3.52, 95% CI 2.76–4.48; *p* < 0.001), and teaching hospital (OR 1.91, 95% CI 1.53–2.40; *p* < 0.001). Conversely, a previous coronary artery bypass graft was associated with a decreased risk (OR 0.26, 95% CI 0.14–0.49, *p* < 0.001). Of the patients who underwent TV-TP therapy, 37.9% received a subsequent PPM during the hospital stay and the mean length of hospital stay was 7.4 days. The results are summarised in Tab. 4.

**Table 4:** Summary of recent large observational study on transvenous temporary pacing (*TV-TP*) therapy in the United States

Summary of study characteristics and results	
First author	Metkus TS
Journal	Chest
Year of publication	2019
Number of patients (*n*)	360,223
Year of inclusion	2004–2014
*Complications linked to TVTP*	
Pneumothorax	0.9%
Pericardial tamponade	0.6%
Non-pericardial bleeding	2.4%
Hospital stay (days)	7.4 ± 0.06
Permanent pacemaker insertion	37.9%
In-hospital mortality	14.1%
TV-TP indication	No data available
TV-TP access route	No data available

## Discussion

This systematic scoping review demonstrates that TV-TP has a wide variety of indications, but is most commonly used for patients with symptomatic atrioventricular block. The femoral vein was the most frequently used access site for TV-TP. Complication rates following TV-TP therapy remain high despite the experience gained over time, yet display a wide range amongst studies potentially related to specialist experience and quality of imaging systems used during implantation. Almost two-thirds of the patients who initially require TV-TP therapy develop a permanent need for cardiac pacing.

The published studies show a heterogeneous patient population with varying indications for TV-TP therapy. The vast majority required TV-TP because of symptomatic atrioventricular block. Severe atrioventricular block may result in haemodynamic instability and syncope and may represent a life-threatening emergency [47]. TV-TP may re-establish normal haemodynamics that are acutely compromised in this setting [12]. There was a relatively large number of patients requiring TV-TP in the setting of acute myocardial infarction. However, the need for cardiac pacing after myocardial infarction has declined over the past decades due to improved therapies, such as revascularisation strategies with thrombolysis and angioplasty [48]. Of note is that in 11.6% of cases the indication for TV-TP was acute myocardial infarction without further specification of underlying rhythm. It is possible that some of these cases can also be attributed to atrioventricular block, reiterating that to be the most important indication.

The most frequently used access route for TV-TP was the femoral venous approach. This might be explained by the ease of advancing the lead to the heart and the reduced chance of complications such as pneumothorax. The best approach to the major venous access site is subject to debate. The different venous routes are associated with specific problems including lead stability, infection, haemorrhage, pneumothorax and patient discomfort [12]. The femoral insertion site has been associated with higher infection rates than the subclavian site, yet with equal rates compared to the jugular access site with intravascular catheters [49]. The use of chlorhexidine for skin disinfection has been shown to be superior to either povidine-iodine or 70% alcohol in reducing bacteraemia [50]. Although less prone for infection, the subclavian insertion site has been shown to be more likely to cause thrombosis compared to the jugular site, especially considering the potential need for a future PPM, also utilising the subclavian vein as an access route. Also, the risk of pneumothorax is higher with this access site compared to the other sites [50]. TV-TP is commonly performed in an emergency setting; hence the choice of approach is often based on individual experience [6, 51]. Femoral placement of the pacemaker could confer benefit in the presence of thrombocytopenia and/or coagulopathy, as pressure can be easily applied and haemostasis achieved in the case of bleeding. Conversely, femoral placement may be accompanied by the least stable wire position and may restrict the patient’s mobility by requiring a horizontal position [12]. Guidelines from the British Cardiac Society recommended the right internal jugular route as most suitable for the inexperienced operator, since this is the most direct route to the right ventricle; this route has had high success rates and few complications [7, 52]. In patients receiving or likely to receive thrombolytic treatment, the brachial and external jugular veins could be considered in addition to the femoral vein as the routes of choice because of the potential for compression in the case of bleeding. If the probability for subsequent PPM implantation is high, it is recommended that the left subclavian approach be avoided, as this is the most frequently used route for PPM implantation [12].

The standard approach for emergency placement of a temporary transvenous pacemaker is utilising fluoroscopic guidance. The necessary transfer to a catherisation laboratory for this procedure and the delay until therapy could be life-threatening in a haemodynamically instable patient. Echography-guided implantation of TV-TP leads could result in shorter times to therapy and avoid the risks of an emergency transfer. Two observational studies reported the feasibility of using echographic guidance in temporary transvenous pacemaker placement in a total of 130 patients [45, 46] and showed a significant reduction in total complications in the larger study (6.8% vs 20.7%, *p* = 0.03) [45]. Also, the median time from decision to active pacing was significantly shorter for the echography-guided group (22 vs 43 min, *p* < 0.01). Although these studies show positive initial results, the results need to be interpreted with caution due to their non-randomised nature, selection bias and relatively small number of patients. Larger randomised studies are needed to assess the full extent of the potential advantages of echography-guided temporary transvenous pacemaker implantation.

To our knowledge, this is the first evaluation of the trend of complication rates following TV-TP since its introduction. The mean reported complication rates have remained high (23%) over the past decades, but have shown a significant decline since its introduction. An analysis performed in the NIS database in the United States between 2003 and 2014 included 43,472 patients and showed an increasing trend in TV-TP-related periprocedural complications up to 17.7% in 2014 (Rozen et al., unpublished data). An even larger study reporting on outcomes of TV-TP in the United States included over 350,000 patients and used the same NIS database for the analysis [16]. Although only potential complications could have been identified, a lower rate of cardiac perforations was observed (0.6%) in this large cohort. Interestingly, the incidence of this life-threatening complication has shown a rise over the last decade. Moreover, the rate of pneumothorax was higher than found in our analysis (0.9%). Non-cardiac bleeding accounted for a complication in 2.4% of patients, which is similar to the rate we found in the most recent decade. The use of echographic guidance might result in safer procedures for the implantation of a temporary transvenous pacemaker, but the risk of bleeding after insertion still remains. The total number of complications found by Metkus et al. was strikingly low at 3.9% [16]. However, there are some important limitations to consider: no data were available on the implant procedure, and complications with no available ICD codes could have been missed (e.g. malfunction of TV-TP therapy, fever, blood-based infection, delirium). This might have resulted in an underestimation of the total complication rate. In our study the complication rates differed substantially between published studies. For instance, Shah and Awan [44] observed a complication rate of 0.8% compared to 80.5% reported by Austin et al. [8]. There are some important aspects in the interpretation of the differences in complication frequency that merit emphasis. First, different inclusion criteria were used, since TV-TP therapy is implemented in a heterogeneous patient population. Second, the definition of complications is different between published articles. Hospital admission following TV-TP was long, with a mean of 11.2 days. These patients are at risk for thromboembolic events, pneumonia and delirium [12, 53]. This may result in a prolonged hospital stay, possibly resulting in an economical and logistic burden.

The high mortality rate of 14.5% indicated a sick patient population in whom TV-TP therapy is required, often to treat life-threatening situations. The mortality rate in the large US cohort (14%) was similar to that in our analysis [16]. Another recent large study (*n* = 4838) by Ng et al. showed a similar mortality rate of 11.8% during the index admission, and a strikingly high mortality rate of 53.6% during over 4 years of follow-up [54]. In this cohort weekend admission was associated with increased mortality compared to weekdays (hazard ratio 1.15, 95% CI 1.06–1.26, *p* = 0.002) and independently predicted all-cause mortality.

The need for a permanent pacing system after TV-TP therapy was high with a mean of 64.2%. A similar number of PPM implantations after TV-TP was found by Rozen et al. (unpublished data): 61.9%. These patients have to undergo two procedures and are therefore at risk for associated complications related to temporary and subsequent permanent cardiac pacemaker implantation. Patients with a TV-TP before PPM implantation are up to 2.5 times more prone to develop an infection [12]. Therefore, the current guidelines state that TV-TP should be avoided, and if necessary should be applied as briefly as possible [12].

These findings underline the necessity for alternative treatments for patients requiring TV-TP. New technologies, for example retrievable leadless pacemaker (LP) systems [55, 56], could provide an alternative for selected patients who require TV-TP therapy. LP therapy has been introduced to reduce complications related to conventional PPM therapy and does not require the use of transvenous leads, likely avoiding lead-related complications associated with TV-TP and conventional PPM. In addition, LP may be an alternative approach in patients who require TV-TP and subsequent conventional PPM implantation without venous access, or who have a history of recurrent device infections [57, 58]. However, as the LP is currently only available as a single-chamber right ventricular device, this approach could potentially be limited to a single-chamber pacemaker population. Future concepts, such as leadless VDD resulting in atrioventricular synchronous pacing [59, 60], might broaden this patient population.

A prospective study on TV-TP complications and outcome is recommended. As the majority of TV-TP therapy patients require a PPM at a later stage and complication rates are high, upcoming techniques such as leadless pacing should be monitored closely as they might prove an alternative to TV-TP therapy and all the possible complications it entails.

### Limitations

The lack of high-quality evidence resulted in the choice to perform a scoping review, but it comes with several limitations: most importantly the inability to perform a meta-analysis of the data. With the aim of providing insight into the current performance of TV-TP therapy we compiled a comprehensive descriptive overview. Due to the heterogeneity of the patients and study designs the conclusions derived from this review are restricted. Definitions of complications as well as patient characteristics differed between published studies. It is important to recognise possible under-reporting of complications (i.e. delirium, pneumonia in the elderly) as these complications might contribute to a poorer outcome and prolong hospitalisation even further. As it was not possible to differentiate asystole during insertion from postoperative asystole this was excluded as a serious complication. For these reasons, reported (serious) complication rates in this overview may be underestimations.

## Conclusion

The most important indication for TV-TP is atrioventricular block; however indications vary widely. The most frequently used access route for TV-TP was the femoral vein approach. Since the introduction of TV-TP therapy its reported complication rates have decreased, but have remained high over the past six decades. PPM therapy following TV-TP is required in the vast majority of patients and therefore alternative treatments, such as LP therapy, could prove a viable treatment in the future.

## Caption Electronic Supplementary Material

Supplement 1: Search strategy
