# Supplementary material for: A comprehensive scoping review on transvenous temporary pacing therapy
Source: Neth Heart J. 2019 Aug 7;27(10):462–73. doi: 10.1007/s12471-019-01307-x (PMC6773795; doi:10.1007/s12471-019-01307-x)
Supplement: Supplementary file 1 — Supplement 1: Search strategy [file 12471_2019_1307_MOESM1_ESM.docx]

**SUPPLEMENTAL MATERIALS**

Supplement 1: Search strategy

Database(s): Ovid MEDLINE(R) In-Process & Other Non-Indexed Citations and Ovid MEDLINE(R) 1946 to Present 
Search until 15-02-2019

| # | Searches | Results |
| --- | --- | --- |
| 1 | ((temporary or temporarily or provisional* or (bridging not ((heparin* or warfarin or enoxaparin* or anticoagulant*) adj3 bridging)) or not-permanent* or nonpermanent* or non-permanent*) adj9 (pacing or pacemaker* or pacer or (lead* adj2 (implant* or insert*)) or ((epicard* or ventricular or atrial or bipolar or unipolar or double) adj3 wire*))).tw,kf. | 2130 |
| 2 | (temporal adj2 pacing).tw. and ((card* adj2 pacing) or pacemaker* or pacer*).mp. | 7 |
| 3 | (TEPW or TEPWs or TPM or TPMs or (temporary adj2 (PM or PMs))).tw,kf. and (wire* or pacing or pacer* or pacemaker*).mp. | 22 |
| 4 | (wait* adj6 (pacing or pacemaker* or PPM or PPMs)).tw,kf. | 15 |
| 5 | ((prophylactic or preventive) adj4 pacing).tw,kf. | 159 |
| 6 | ((prophylactic or preventive) adj (pacemaker* or pacer*)).tw,kf. | 58 |
| 7 | or/1-6 [ TP-I ] | 2325 |
| 8 | Cardiac Pacing, Artificial/ | 20221 |
| 9 | Pacemaker, Artificial/ | 25039 |
| 10 | ((artificial or cardiac or cardial* or endocardia* or epicardia*) adj6 (pacing or pacemaker* or pacer*)).tw,kf. | 11714 |
| 11 | or/8-10 [cardiac pacing] | 44860 |
| 12 | (temporar* adj6 (lead or leads or BiVP or paced or resynchron* or wire or wires or DHBP or HBP or device*)).tw,kf. | 2242 |
| 13 | 11 and 12 [ TP-II ] | 378 |
| 14 | 7 or 13 [ TP-I & TP-II ] | 2369 |
| 15 | (exp animals/ not humans/) or (rat or rats or mouse or mice or murine or rodent* or dog or dogs or bitch* or beagle or beagles).ti. | 4786093 |
| 16 | 14 not 15 [human studies on temporary pacing] | 2292 |
| 17 | meta-analysis/ or (meta analy* or metaanaly* or meta?analy*).tw,kf. or ((systematic* adj3 (review or literature or evidence or search*)) or ((summari* or review) adj3 evidence) or (search* adj12 (literature* or ((electronic or medical or biomedical) adj3 database*) or exhaustive)) or medline or pubmed or embase or psychinfo or (CENTRAL and cochrane) or "Central Register of Controlled Trials").tw. or (cochrane or clinical evidence or EBM).jw. [SECONDARY STUDY-filter] | 367436 |
| 18 | 16 and 17 [secondary studies on temporary pacing] | 19 |
| 19 | (expert or current or cochrane or clinical evidence or EBM).jw. or editorial/ or books/ or (case report or "one case").ti. or (case reports/ not (review.pt. or (serie* or (review adj3 case*)).tw.)) or (systematic* adj3 (review or literature)).ti. or ((search* adj12 (literature* or ((electronic or medical or biomedical) adj3 database*) or exhaustiv* or systematic*)) or medline or pubmed or embase or psychinfo or (CENTRAL and cochrane) or "Central Register of Controlled Trials").tw. ~~or (cochrane or clinical evidence or EBM).jw.~~ or ((review/ or meta-analysis/ or (meta analy* or metaanaly* or meta?analy*).ti,ot,kw. or (systematic* adj3 (review or literature)).tw,kw. or review.ti. or comment/ or technical report/) not (exp clinical trial/ or exp cohort studies/ or major clinical study/ or case reports/ or ("own study" or "own experienc*" or "own case*" or ((review adj2 (case or cases)) or (case* adj3 serie*))).tw.)) [PRIMARY STUDY FILTER] | 5482262 |
| 20 | 16 not 19 [individual studies on temporary pacing] | 1398 |
| 21 | remove duplicates from 20 | 1397 |
